# Pasta-Making Process: A Narrative Review on the Relation between Process Variables and Pasta Quality

**DOI:** 10.3390/foods11030256

**Published:** 2022-01-19

**Authors:** Andrea Bresciani, Maria Ambrogina Pagani, Alessandra Marti

**Affiliations:** Department of Food, Environmental and Nutritional Sciences (DeFENS), Università degli Studi di Milano, Via G. Celoria 2, 20133 Milan, Italy; andrea.bresciani@unimi.it (A.B.); ambrogina.pagani@unimi.it (M.A.P.)

**Keywords:** pasta making, pasta, hydration, extrusion, drying, cooking quality

## Abstract

Pasta is an increasingly popular food worldwide and different formulations have been developed to improve its nutritional profile. Semolina that is high both in protein and gluten content is recognized as the ideal raw material to produce conventional dry pasta. When alternative raw materials are used, an understanding of the relationship between processing variables and pasta quality is crucial in order to optimize the redesign of the production process. This review aims to: (1) investigate the main challenges of the pasta-making process, highlighting the processing variables that most affect pasta quality; and (2) indicate the unknown factors that influence the pasta-making process and which need to be studied. After overviewing the last twenty years of research in the pasta sector, the interplay/relationship between processing variables and pasta quality is examined, together with the main innovations proposed for each step of pasta processing. An analysis of all the variables involved in the process and their influence on each other will elucidate how to optimize certain parameters to ensure the production of pasta with the desired characteristics.

## 1. Introduction

Pasta is one of the most common and popular staple foods thanks to its sensory and nutritional value, convenience, and versatility [1]. It is reported that about 14.3 million tons of pasta are produced annually worldwide. The main producer is Italy, followed by the United States, Brazil, Turkey, and Russia. Italians are the main pasta consumers, with 23.1 kg per capita per year, followed by Tunisians (17 kg), Venezuelans (12 kg) and Greeks (11.4 kg) [2]. According to Italian law, “dried pasta” must be produced with water and durum wheat (*Triticum durum* Desf.) (i.e., semolina, coarse semolina, or wholemeal semolina) [3]. Although in the rest of the world (except for France and Greece) common wheat (*Triticum aestivum* L.) can be used for pasta production, it is well-known that only durum semolina can assure the best product quality, in terms of dough rheological properties, cooking quality and consumer acceptance [4,5]. However, it should be noted that common wheat is approximately 20–25% cheaper than durum wheat, making it an interesting raw material for worldwide production thanks to its high availability and (cost-effectiveness/relatively low cost [6].

Pasta plays a key role in the Mediterranean Diet. WHO (the World Health Organization) and FAO (the Food and Agriculture Organization of the United Nations) described pasta as a healthy, sustainable, and quality food model. Moreover, in 2010, UNESCO (United Nations Educational, Scientific and Cultural Organization) declared pasta an intangible cultural heritage of humanity [7]. One of the main reasons for the success of pasta is its nutritional profile. Indeed, pasta generally is very nutritious, due to its low amount of fats and readily digestible carbohydrates [8]. Moreover, pasta can supply healthy components, such as fibre or prebiotics [9,10]. The low cost and long shelf life of pasta make it popular with many diverse groups of consumers [11].

Despite being considered a traditional product, pasta (and the pasta sector in a broader sense) has been able to evolve over the years to meet the needs of the market that has expanded from Italy throughout the world through the improvement of production efficiency, on the one hand, and the enhancement of product quality from hygienic, sensory and nutritional stand points on the other. The above-mentioned aspects are the driving force behind pasta innovation. The various references present on the market—including wholegrain, multigrain, gluten-free, pulse and vegetable-enriched pasta—are examples of product innovation. Consumers certainly appreciate the taste and cooking behavior of semolina pasta [12] and the healthy features of fiber-enriched pasta [13]. However, what consumers ignore are the challenges of producing these kinds of products, the know-how and processing innovation behind each package of pasta. The change of a single variable—such as the type of raw material (refined vs. wholegrain semolina)—can affect the entire process and product quality. In this context, it is important to single out the current factors (i.e., what process variables are affected by alternative raw materials) in order to adapt the process properly in order to obtain a high-quality end product. This review focuses on individuating the main process variables that influence the quality of the product. Understanding the relationship between processing variables and pasta quality is essential in “redesigning” the process when alternative raw materials (i.e., ingredients other than durum wheat semolina) are used. 

The present review is divided into three sections. Firstly, we provide an overview of research on pasta and the pasta-making process carried out in the last twenty years. Secondly, for each step of pasta processing, the interplay between the main variables in affecting and determining the quality of the final product is discussed, together with the main innovations published in research articles. Finally, the last section focuses on the main knowledge gaps of the sector (i.e., how to produce pasta from alternative raw materials), with the hope of stimulating further study in this field. 

## 2. Overview of Research on Pasta

A search using “pasta” or “spaghetti” as keywords (to be searched in the title of documents) was carried out on the Web of Science database. More than 50% of the research articles published in the Food Science and Technology category were published in the last 10 years, with a progressive increase in number over the years and an average of 80 contributions per year over the last five years. 

There are numerous reasons that explain this trend which, among other things, coincide with the reasons that accompanied the transition of this food from a “traditional Italian product” to a “product of international success” [14]. Pasta products are popular due to their simplicity in terms of formulation (they can be prepared with only two ingredients: semolina (from durum wheat) or flour (from common wheat) and water), the technological process involved (it is a continuous process, completely automated and consisting of few operations) and methods of preparation by the consumer. Dry pasta is also characterized by long shelf life, up to three years, thanks to its low humidity (generally lower than 12.5%), as well as its great adaptability to different tastes and traditions. In addition, in the presence of a vegetable or meat- or fish-based condiment, it represents a complete and balanced dish from a nutritional point of view, with a medium–low glycemic index [15]. This last characteristic is due to the technological process that leads to the formation of a compact final structure that is slowly accessible to digestive enzymes [16,17]. 

**Figure 1:**
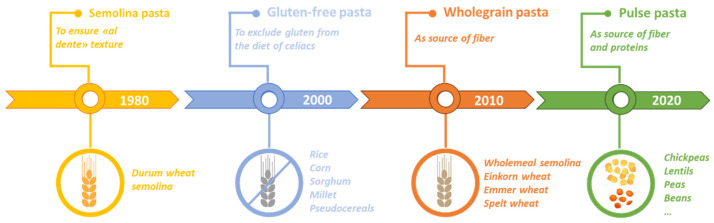
Evolution of types of pasta and the related raw materials.

Most studies focus on pasta formulation, including flours from grains other than durum wheat (or their fractions) or other ingredients (including vegetables) to improve the nutritional profile of the pasta [11,18,19,20,21,22,23]. Consumer interest in different types of pasta reflects an evolving market trend (see Figure 1) to obtain certain nutritional benefits deriving from the specific alternative raw materials used in pasta production. 

Strategies and opportunities for producing functional pasta have been widely reviewed in the last ten years [11,18−23]. What these studies have in common is the awareness that pasta can be considered an important and interesting carrier for bioactive compounds, especially dietary fiber. For example, a portion of 80 g of whole wheat pasta provides up to 6 g of the recommended daily 25 g of dietary fiber for those with energy intakes of less than 2000 kcal/day [24]. From the literature, it emerges that the main aim of researchers is to identify the maximum level of fiber (or source of fiber) enrichment possible in order to benefit from a nutritional standpoint, without compromising the quality of the final product in terms of cooking quality and sensory profile. Overall, the quality of enriched pasta is generally similar to that of traditional pasta for enrichment levels of less than or equal to 10% [20]. For higher levels, quality can be significantly lower, suggesting the need for further studies to optimize the pasta-making processes of fiber-enriched pasta. 

**Figure 2:**
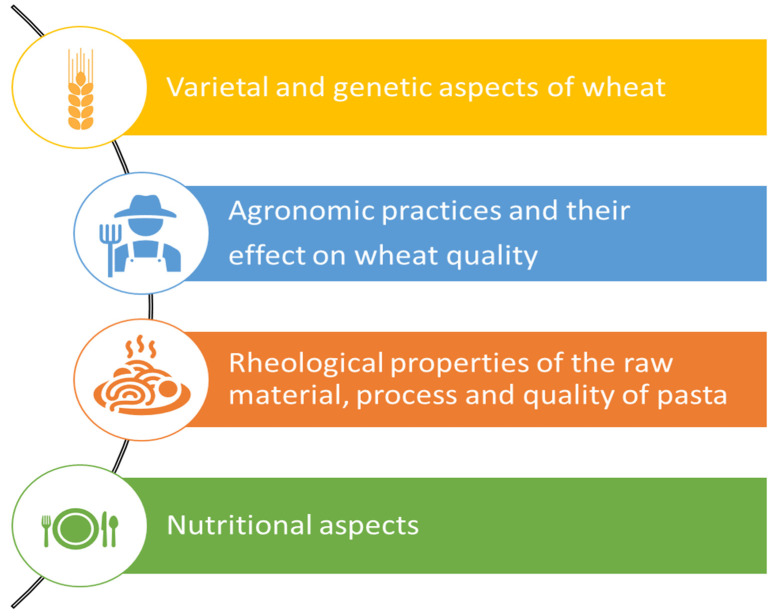
The main topics related to durum wheat and semolina pasta in the last 40 years.

In a recent study, Cecchini et al. [25] elaborated the results of a literature search through VOSviewer software using the Scopus database and the keywords “quality and pasta” and “quality and durum wheat”. Compared to our search, our colleagues have, on the one hand, limited the search to pasta from durum wheat and, on the other, extended it to the quality of the raw materials. Using these criteria, about 2000 studies were published on pasta from 1987 to 2018, dealing with the following topics (Figure 2): (1) varietal and genetic aspects of wheat; (2) agronomic practices and their effect on wheat quality; (3) rheological properties of the raw material, process, and quality of pasta; (4) nutritional aspects. Specifically, the latter seem to have gained more interest in recent years compared to topics related to genetics and breeding. Although the contribution of Cecchini et al. [25] mapped the evolution of durum wheat and pasta quality research topics, it did not provide insights into either the relation between processing conditions and pasta quality or the recent advances in the pasta-making process, which are the objectives of the present review. 

## 3. Overview of Research on the Pasta-Making Process

As regards processing, pasta-making is a continuous process, consisting of three main steps: dosing and mixing, kneading and shaping (by extrusion or sheeting), and drying. Despite the vast amount of bibliographic information on pasta, the debate over the question “Does the raw material or the pasta-making process matter more?” is still ongoing, also considering that wholegrain pasta is becoming more and more popular. 

It is well known that durum wheat semolina characterized by high protein content and strong gluten—able to withstand the physical stress occurring during extrusion, drying, and cooking—is the ideal raw material for high-quality pasta [4]. However, even starting from good-quality semolina, the production of good-quality pasta is not ensured if each step of the continuous pasta-making process is not properly carried out. Table 1 summarizes the aim of each step of the pasta-making process, together with the intrinsic and extrinsic parameters affecting the dough and/or pasta. It is worth noting that the pasta-making process from gluten-free raw materials is reported elsewhere [26,27,28,29]. 

So far, the effect of each step of the pasta-making process has been evaluated with respect to its impact on pasta structure and quality [17,30,31,32]. On the other hand, the effect of processing variables (i.e., hydration level, extrusion pressure/temperature/mechanical energy) on pasta quality has not yet been exhaustively investigated. Based on these considerations, the role of the main variables—involved in each step of the pasta-making process—on pasta quality will be discussed in the following sections.

### 3.1. From Dosing to Mixing

In the first step of pasta-making, semolina and water are carefully dosed and blended together to form a hydrated mixture with a total moisture content of about 30–32%. The amount of water added to semolina (27–29 g/100 g) is far from the hydration level used in bread-making (50–60% water absorption, namely 45–50% moisture), which is essential for promoting the even water dispersion inside the solid mass. In other words, in the pasta-making process, hydration ensures the correct solvation of proteins while gluten is only partially developed at this stage. Only appropriate protein hydration will assure—in the following steps—the formation of a continuous gluten network capable of restricting and preventing excessive starch swelling during cooking. 

Besides the amount of water, other factors may affect semolina hydration and thus the physical properties of pasta and its quality. Among them, protein, ash, fiber, and damaged starch content, as well as particle size (Table 1). Semolina samples with low ash and damaged starch content result in a dried product characterized by an amber-yellow color, low brown specks (due to the presence of bran particles), and low heat damage [30]. Low ash content is assured by a medium–low extraction rate (60–65%), whereas low-damaged starch is assured by milling durum wheat to large particle sizes (>400 μm). However, the choice is not so easy, because a medium extraction rate is synonymous with a low milling yield (and thus productivity), whereas a large particle size might result in low hydration kinetics with inadequate moistening of semolina. A regular and even protein structure is essential to guarantee a product with good cooking behavior, resulting in high firmness and absence of stickiness and bulkiness [33]. 

Particle size also plays a key role in wholewheat semolina. A positive correlation between the geometric mean diameter of flour particles and the cooking behavior of wholemeal semolina pasta (i.e., high firmness and low cooking loss) was assessed. At the same time, broad particle distribution negatively impacts pasta cooking quality [34]. As regards reconstituted semolina/bran blends, bran particle size doesn’t seem to impact on pasta cooking behavior but the semolina/fine bran blend is preferred since the resulting pasta showed higher mechanical strength than pasta from the semolina/coarse bran blend [34].

### 3.2. The Effect of Hydration on the Extrusion Process and Pasta Quality

The amount of water added to semolina and its uniform dispersion inside the mass are critical parameters because mistakes made in this first operation can hardly be corrected in the following steps of pasta-making. In the case of uneven hydration (often caused by limited amounts of water), the final product may form the characteristic white spots which indicate a potential weak structure and decrease the quality of the product in terms of both appearance and texture. On the contrary, excessive hydration results in a sticky product, with low mechanical resistance and poor cooking quality [35]. 

De la Peña and Manthey [36] evaluated the effect of different levels of hydration (from 30 to 34%) on the extrusion properties of refined or wholemeal semolina (alone or in combination with flaxseed flour) and on the cooking behavior of the respective pasta samples. The results of this study showed that specific mechanical energy (SME) and extrusion pressure decrease as the level of hydration increases. Specifically, as regards the extrusion pressure, the formulation of pasta seems to have a significant effect: the semolina dough registers a decrease in pressure lower than that observed for wholemeal semolina. The plasticizing action of water facilitates the handling of the mixture inside the extruder, reducing the extrusion parameters. A correlation was highlighted between the viscosity of the dough (measured by a capillary rheometer) and the parameters of extrusion pressure, as well as mechanical energy [37]. Specifically, increased hydration promoted a decrease in the apparent viscosity of the dough without increasing the extrusion rate. Moreover, high levels of hydration (32–34%) are associated with a reduction in brightness/luminosity and an increase in the degree of red (a*) but do not affect the degree of yellow (b*) [37]. 

As a result of the decrease in extrusion pressure and mechanical energy, the diameter and density of the spaghetti decreases as hydration increases. Since dough at 30% hydration shows high consistency and resistance to flow, it was hypothesized that these systems could bring high pressure to bear on the Teflon coatings of the die inserts, compressing them and thus resulting in an increase in the diameter of extruded spaghetti [37]. On the contrary, formulations hydrated at 34% level, showing lower consistency, exert a lower pressure during extrusion by reducing the diameter of the spaghetti. It is also possible that spaghetti produced with the highest hydration levels (and which is therefore heavier) may have been slightly stretched on the reeds during drying. The smaller diameter in the 34% hydration formulations seems to be responsible for the reduced hardness of the cooked pasta and the greater cooking losses, as a result of the faster migration of water towards the core of the spaghetti [37]. The role of porosity/compactness as a consequence of the decrease in extrusion pressure should also be considered.

### 3.3. The Effect of Formulations on Hydration Levels

The hydration operation is even more critical when grains other than durum wheat or ingredients are included in the formulation. Re-formulating pasta makes it essential to study and optimize the level of hydration as this affects not only the characteristics of the dough (in particular its processing during the extrusion phase) but also the quality of the final product. Fiber, due to its high hydrophilicity, competes with proteins for water absorption; it could, therefore, reduce the water available for their solvation, compromising the formation of a uniform network. Furthermore, the worsening of pasta quality in the presence of fiber is due to the dilution effect of gluten (as a result of the lower amount of semolina in the formulation), as well as a discontinuity in the protein network caused by the interference of the non-starch polysaccharides [38]. 

Considering the effect on the formulation, wholemeal semolina doughs showed higher viscosity, even at high levels of hydration (34%), compared to the reference sample (refined semolina). The bran fractions in the wholemeal sample would require more water to show the same rheological behavior as the reference sample [37]. Similar results were found in the presence of flaxseed flour, buckwheat bran or durum wheat bran [39]. However, based on the effect of hydration levels on extrusion parameters, the study of de la Peña et al. [37] states that hydration should not exceed 32% for semolina, whole wheat semolina and their blends. This level should be reduced to 30% in the case of formulations rich in lipids, such as flaxseed flours [37]. Lipids, in fact, can have a plasticising effect on the dough and act as a lubricant by reducing the friction generated inside the extrusion cylinder, thus reducing extrusion pressure, mechanical energy and SME [37].

A possible solution to limit the competition for water between fiber and proteins could be to hydrate the two ingredients separately (for example, semolina and bran) before extrusion, as proposed by La Gatta et al. [40]. From a sensory point of view, separate hydration seems to have a positive effect on color and the resistance to breaking of uncooked pasta and on the elasticity, firmness, adhesiveness and bulkiness of cooked pasta. In addition, a decrease in cooking losses was measured. This approach would allow semolina proteins to solvate and interact optimally by limiting the interference caused by the fiber, forming a structure capable of retaining the swelling and solubilization of starch during cooking. This approach would produce better-quality pasta while maintaining suitable hydration levels for the extrusion process. However, despite the encouraging results obtained on a laboratory scale, the scale-up of the process remains to be investigated. 

### 3.4. New Trends in Hydration Systems

Since hydration is mostly influenced by the physicochemical characteristics of the raw material (Table 1), to ensure correct protein hydration, raw materials with a low extraction rate should be preferred (such as semolina obtained from the innermost part of the endosperm, for its low ash and fibre content) and with low starch damage, therefore medium-to-large sized semolina particles. According to many pasta producers, semolina with particle sizes ranging from 250 and 450 μm seems to guarantee homogeneous hydration. However, a large particle size (more than 450 μm), highly appreciated by some Italian pasta-makers for the low starch damage, makes it difficult to hydrate the semolina particles correctly, promoting the formation of white spots. In this context, besides accurate devices for the dosing step, various hydration systems have been proposed to guarantee a more homogeneous hydration of the raw materials. Indeed, at the end of the mixing step in the conventional extrusion press, dough appears as “lumps” of different sizes. In the innovative devices, the premixing and mixing steps are usually combined in a single operating unit. Among the proposed systems, the Polymatic press (Bhuler, Uzwil, Switzerland) mixes and develops pasta dough in 20 s. A twin-screw extruder forms the dough, which is directly sent to the extruder. The entire system is under vacuum, which assures excellent pasta color. Other advantages of this system include the rapid changeover of dies, which helps when different forms of short pasta are being manufactured, as well as a clean-in-place system for excellent sanitation [41]. 

Among the other solutions suggested to improve the initial steps of the pasta-making process, the effects of the innovative Premix^®^ and Bakmix^®^ mixing systems (Storci S.p.a., Collecchio, Italy) were compared with a conventional system (V50, Storci Spa) [42]. The centrifugal force applied in the Premix^®^ would promote the rapid (1–2 s) and uniform hydration of the surface of each individual semolina particle, followed by a rest phase (10 min) before extrusion. In the Bakmix^®^ system, hydration is divided into two phases: 2 s in the Premix^®^ system and 18 s in an extruder operating at low pressure (about 10^6^ Pa). All the mixing processes result in products (fresh pasta obtained by two shaping approaches: extrusion or lamination) of acceptable quality and characterized by good cooking behavior, with cooking loss values lower than 3 g/100 g pasta.

In general, the new systems facilitate a more uniform distribution of water throughout the flour compared to traditional mixing but in a significantly shorter time; therefore, a well-developed protein matrix may not be formed [42]. This results in a pasta dough that is less extensible and more resistant to deformation, characteristics considered to be negative for fresh pasta. The authors of the work suggest that, due to the short mixing time, it may be necessary to increase the level of hydration to obtain a better-quality product. However, this theoretical solution does not seem to be the best for obtaining a good-quality pasta, as stated by Manthey et al. [43] and already discussed. Moreover, their study focused on understanding the effect of the different hydration systems on the characteristics of fresh pasta; it would therefore be interesting to re-propose this experimental plan completing the pasta-making process with drying; in fact, drying could reduce the differences highlighted in fresh pasta by Carini et al. [42]. Moreover, the effect of non-traditional hydration systems might be particularly successful when applied to wholegrain semolina. 

In the new pasta-making plans, the pre-mixer system is connected to a stabilization belt mixer (Beltmix^®^; Storci S.p.a., Collecchio, Italy), in place of the traditional shaft and blade mixing tank. The belt mixer consists of a slow-moving conveyor belt. Since the dough is not subjected to any kind of mechanical action, the system drastically reduces the oxidation of the raw materials, which maintains the bright yellow color of semolina. In addition, compared to the traditional mixer, the belt mixer is easier and faster to clean, as stated by the device manufacturer. 

### 3.5. From Kneading to Shaping 

Shaping or forming aims at creating a well-defined shape (Table 1) and represents the heart of the pasta-making process. It can take place in two ways, by extrusion under pressure or by roll-sheeting. The former involves the kneading of the dough into a cylinder through a screw that compresses and pushes the mass towards the die, where pressure can reach values of 10 MPa or more. The size and the design of the screw can vary according to the manufacturing companies. Generally, screws are divided into three sections: the feeding section where the “lumps” of dough are pushed towards the transfer section and then to the extrusion section. During this flow, the dough undergoes a spiral movement favoring the kneading. At a macroscopic level, the mass acquires compactness, but the gluten network can undergo stretching and stresses of high intensity, especially in the final section of the extruder, before the dough passes through the die [44]. The second approach used to shape the dough involves rolling the dough through passages in cylinders that gradually and lightly reduce the thickness of the dough until a sheet of the desired thickness is obtained. During sheeting, dough is subjected to pressure for a very short time, i.e., only when it passes into the gap between the two cylinders; then the dough can immediately relax and recover from the deformation. Of the two processes, extrusion is the preferred approach at an industrial level not only for its higher productivity but also for its versatility; through extrusion, in fact, more than 200 different pasta shapes can be obtained. For this reason, the extrusion process is more studied than lamination. Indeed, most of the analyzed works focus exclusively on the study of some variables of the extrusion phase or on the comparison between extrusion and lamination.

The use of unconventional raw materials and/or incorrect hydration of dough affect this operation, as the variables involved during shaping are greatly influenced by the amount of water in the dough, which, as discussed in the previous paragraph, must be optimized based on the physicochemical characteristics of the raw material, including particle size, content of damaged starch and presence of fiber. 

If the hydration step affects extrusion, the latter can irreversibly break down the protein network, resulting in its disruption during cooking especially when poor-quality raw materials are used. At the same time, improper extrusion conditions can cause starch swelling and gelatinization, due to the heat generated by shear stress. These setbacks can be limited by keeping the extrusion temperature below 50 °C and selecting semolina varieties having high starch gelatinization temperatures to delay starch swelling and solubilization and to decrease interference with protein reticulation [45,46].

### 3.6. The Effect of Extrusion Variables on Pasta Quality

Among the extrusion variables, the pressure (measured in the final part of the extrusion cylinder) and SME are useful for evaluating the overall process. They are correlated with and influenced by the same variables, including the level of hydration, the speed of the screw and the extrusion temperature. Since, as is known, the pressure varies during the advancement of the dough along the screw (reaching the maximum value near the die), studies usually consider the SME parameter. Specifically, the focus is on the relationship between hydration level and SME. An overly hydrated dough, being less compact, would require a lower SME and would not pose sufficient resistance, inside the extrusion cylinder, to promote protein aggregation and therefore a satisfying formation of gluten [43]. On a macroscopic level, a low SME, as seen above, reduces the density of spaghetti [36,43]. Water unbound to proteins and other hydrophilic (macro)molecules would be in a free state, making it easier to evaporate during the subsequent drying phase; this phenomenon would reduce density. This hypothesis could be confirmed by the study of the distribution and mobility of water inside spaghetti using NMR techniques [47,48]. 

The variables of the extrusion process (pressure, speed and SME) appear to be unrelated [43] or weakly correlated (r = 0.31–0.44) [36] to the diameter of spaghetti, suggesting that other factors are responsible for the determination of that characteristic. As previously discussed, in addition to hydration, the formulation also influences SME. In particular, the presence of bran or oil seeds reduces SME values; in fact, the presence of lipids helps lubricate the dough on the extrusion screw. As the dough poses less resistance to extrusion, it forms spaghetti with a smaller diameter [36,39]. As reported by de la Peña et al. [36], the diameter of spaghetti inversely affects the amount of material released into the cooking water.

The extrusion temperature also influences the quality of pasta in terms of cooking losses. Indeed, the increase in temperature in the extrusion cylinder from 35 °C to 70 °C leads to an increase in cooking losses up of to 250% [49]. If semolina proteins denature while the mass undergoes mixing and kneading, the denatured proteins are no longer able to interact in this phase with each other to create a protein network capable of retaining the starch granules during cooking. At high temperatures (about 70 °C) during extrusion, the increase in the level of hydration (from 44 to 48%) and in the rotation speed of the screw (from 15 to 30 rpm) has a positive effect on the final characteristics of pasta [49]. The high hydration, combined with the high speed of the screw, in fact, reduces extrusion time, thus limiting the damage that the high temperature could cause to proteins and their ability to aggregate. As is well known, temperatures between 40–50 °C are considered optimal for the pasta-making process of semolina, as they are not associated with significant denaturation of proteins and starch gelatinization but facilitate the extrusion of the dough by decreasing its viscosity. These considerations were also confirmed in the study by Debbouz and Doetkott [35]. Applying an experimental design and considering different levels of hydration (30–32–34%), water temperature (35–45–55 °C), mixing time (3–5–10 min), extrusion temperature (35–45–55 °C) and screw speed (20–25–30 rpm), the authors highlighted how all the variables have a significant effect on pasta quality. The hydration level and the temperature of the extrusion cylinder are the variables with the greatest influence. In particular, pasta cooking losses are reduced at hydration levels between 31.5 and 32% and extrusion temperatures between 45 and 50 °C.

Optimal extrusion conditions vary according to the formulation and how the design of the experimental approach optimizes the process. This holds true for various formulations. For example, for the production of wheat spaghetti enriched with soy flour, the best product (in terms of color and cooking behavior) is obtained when about 57 g of flour, 12 g of soy and 31 g of water are extruded at 35 °C and 40 rpm [50]. In the case of semolina and millet pasta (50:50), the optimal process conditions are as follows: extrusion temperature = 70 °C; hydration level = 30%; extrusion speed: 12 rpm; screw speed/feeding speed ratio = 10 [51]. 

As regards die extrusion, it is known how the coating material of the die inserts affects the appearance of the pasta: Teflon gives the product a smooth and bright yellow appearance, while bronze inserts produce a rough surface [52]. Furthermore, the use of a bronze die has the disadvantages of lowering extrusion pressure and die extrusion speed as well as a more rapid consumption of the part in contact with the dough [53]. Bronze-extruded spaghetti is more porous and therefore more fragile (breaking strength decreases by 20–30%) than Teflon-extruded products [54]. Furthermore, the rougher surface of the bronze-extruded spaghetti, together with its greater porosity, favors the deposition of eggs by *Sitophilus oryzae* (L.) (*Coleoptera Dryophthoridae*) and therefore is a more likely place for insects to incubate compared to Teflon-extruded pasta [54].

### 3.7. Type of Shaping: Extrusion vs. Sheeting

Some research compared the effect of the type of shaping on the structure and quality of the dough. Among these, the study by Zardetto and Dalla Rosa [55] involved fresh pasteurized pasta (76% semolina, 19% egg, 5% water) produced by extrusion or lamination. The results show that fresh pasta obtained by extrusion absorbs more water during cooking and releases a greater quantity of dry substance than pasta obtained by lamination. Extrusion does not form a continuous and homogeneous protein network as occurs for lamination. Furthermore, the mechanical stress exerted by the screw leads to the partial degradation of the starch and probably also to the formation of components (reducing sugars) capable of contributing to the Maillard reaction. In fact, higher furosine levels were found in extruded than in laminated fresh pasta. Pasta obtained by extrusion generally shows higher consistency values than laminated pasta, but cooking reduces the differences between the two types of product, making them more similar. From a molecular point of view, the cooking of extruded pasta promotes the formation of bonds between proteins, an indication that the extrusion process does not lead to the complete formation of a network but to the exposure of thiol groups that interact with each other during the cooking phase. In laminated pasta, on the other hand, the gluten network is well formed, as shown by the high resistance to disintegration (evaluated by sensory analysis) and low adhesiveness (instrumentally evaluated) of cooked pasta [56]. However, the differences observed at the structural level by Zardetto and Dalla Rosa [55] do not imply sensory differences and are probably not perceived by most consumers, as they are probably masked by egg proteins. 

Lastly, the study by Carini et al. [57] compared different shaping processes (extrusion, rolling, and vacuum lamination) using a simpler dough system, consisting exclusively of semolina and water (70:30). The macroscopic characteristics of pasta (color, cooking losses, and firmness) seem to depend on the process, while the water status or how the water interacts with the biopolymers (the ability to retain frozen water and water mobility) was only slightly influenced by processing conditions [57]. Specifically, the extrusion process, due to the greater mechanical stress it requires, seems to facilitate the interactions between water and biopolymers, resulting in a more extensible product. On the other hand, the less stressful conditions for lamination result in a structure that is less compact and less extensible but better able to retain solids during cooking, confirming the results of Zardetto and Dalla Rosa [55]. The application of a vacuum to the lamination process seems to improve the quality indicators of fresh pasta, resulting in a product characterized by a more yellow color and with extensibility and consistency similar to fresh pasta obtained by extrusion. The application of a vacuum during lamination may have eliminated the air contained in the dough as a result of lamination by better compacting the biopolymers and facilitating the interactions between them and those with water.

The different effect of the shaping processes on the quality of pasta is even more evident when using common wheat, as it is less able to stand the physical stresses occurring inside the press. The greater compactness of the structure obtained by extrusion corresponds to longer cooking times and slower water absorption, which, however, does not translate into better cooking behavior for dry pasta. This feature is clearly linked to the different organization of proteins. The gluten network, in fact, appears more continuous in the case of laminated pasta, probably thanks to the lower stress and the action of the rollers that more effectively align the protein fibrils. The result is a firm pasta without stickiness. Finally, in the case of a semolina-based formulation enriched with buckwheat (25%), the preferred technology involves the extrusion of a sheet whose thickness is gradually reduced by rolling [56]. This process, in fact, seems to create a structure that is compact (as suggested by the slower hydration kinetics) and at the same time continuous (as suggested by the slower gelatinization of the starch granules), resulting in a product with lower cooking losses, greater firmness and less of a tendency to disintegrate during cooking.

### 3.8. Drying

Particular attention is paid to the final step of the pasta-making process: the drying step. As is well-known, the drying process gives dry pasta its final characteristics of physical and chemical stability and allows its shelf life to be extended. The overall cooking quality of the final product (high degree of firmness, low stickiness and low cooking loss) is the result of several simultaneous phenomena within pasta, whose extent depends on both raw material characteristics and the temperature–moisture conditions applied during drying. 

The variables that regulate this phase (temperature, relative humidity, and time), in fact, can be modified by proposing various combinations (and as many drying cycles) in order to promote the coagulation of proteins and improve the cooking behavior of pasta (Table 1). In particular, the physicochemical modifications of the main macromolecules control pasta cooking behavior in an opposite way. When protein coagulation in the continuous network prevails, the starch material is trapped within the network and the cooked pasta will be firm with no stickiness on the surface and consequent bulkiness. On the contrary, when the protein network is not strong and elastic enough, the starch swells and gelatinizes before protein coagulation takes place. 

**Figure 3:**
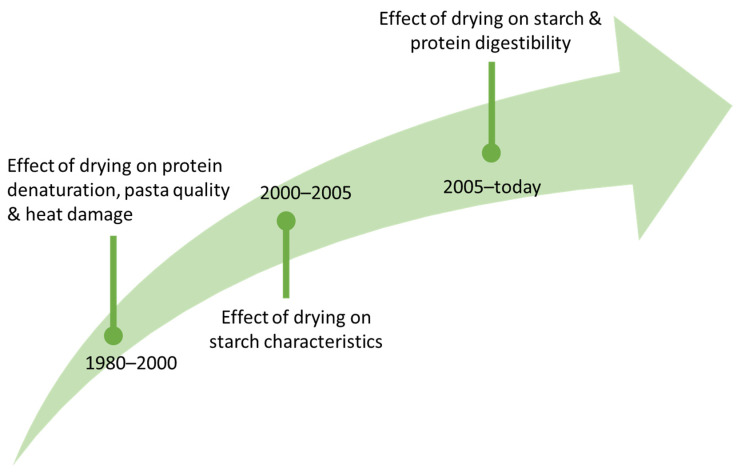
Trends in the research topics on pasta drying over the past 40 years.

Over the years, the scientific community’s interest has changed as summarized in Figure 3. 

The focus of studies shifted from the effects of high- and low-temperature drying cycles on the denaturation of proteins and pasta quality [58,59,60], also in relation to heat damage (1980–2000; see the review by De Noni and Pagani [30]), to the effect of drying on starch characteristics (2000–2005; Padalino et al. [61]), including aspects relating to digestibility (in the last 15 years; see the review by Petitot et al. [17]).

As regards the effect of drying temperature on pasta quality, high temperature drying cycles (>65 °C) are effective in improving the sensory characteristics of pasta [61], especially in the case of pasta made with semolina low in protein [59,62]. The same effect was not evident when pasta made from semolina with strong gluten was prepared [59]. Using multiple regression analysis, D’Egidio et al. [62] showed that stickiness played the most significant role relative to firmness and bulkiness in the case of pasta dried at a low temperature, whereas at a high temperature the three sensory attributes had a similar importance.

Countless studies have addressed the issue of starch digestion in pasta, in view of its relevance to controlling glycemia, but only a few studies have addressed the issue of protein digestion in pasta. Some of these reports have addressed protein-digestibility issues related either to the use of different wheat varieties or to the impact of processing on it [17,63,64,65,66]. However, none of these studies appears to have fully addressed the complexity of the protein pattern in the raw material, as well as the relevance of protein–protein and protein–starch interactions in these complex matrices, either before or after processing. Moreover, conflicting results have been obtained due to the use of different methodologies as well as different pasta-making conditions. A great amount of variation can be seen in the drying conditions (i.e., time, temperature and relative humidity) of published pasta studies, making it difficult to compare findings obtained from various laboratories as pointed out by Murray et al. [32] The meta-analysis work carried out by Mercier et al. [20] on the relationship between the production process and the quality of enriched pasta confirmed what has already been studied for pasta from semolina of various qualities [62]: drying pasta at temperatures above 60 °C can partially compensate for the weakening of the structure of pasta (which is attributed to the enrichment and dilution of gluten) due to the reinforcing effect provided by protein coagulation. Wholewheat spaghetti dried at a low temperature (40 °C) had higher cooking loss but better overall appearance, mechanical strength and cooked firmness than wholewheat spaghetti dried at a high temperature (70 °C) [38]. Similar findings were found when comparing the quality of wholewheat pasta dried at 60 °C or 85 °C: a low temperature was effective in decreasing cooking loss and increasing firmness, even if differences in texture could not be detected using a trained panel [67,68].

Findings on the relation between the products of the Maillard reaction (i.e., advanced glycation end products, AGEs, such as the ε-pyrrole-lysine pyrraline or ε 2-formyl-5-hydroxymethyl-pyrrolaldehyde) and protein digestibility [64,69] as well as the onset of some diseases [70] have brought researchers’ attention back to the investigation of heat damage. Pasta dried at a low temperature had low amounts of furosine, which is the most widely used marker for assessing the extent of the Maillard reaction [27,71]. Many pasta producers stress the importance of drying conditions, specifically the use of slow and/or low-temperature drying cycles. Unfortunately, this terminology is not sufficient to provide clear and univocal indications of pasta quality and/or the intensity of heat damage. The survey carried out on more than 60 pasta samples available on the Italian market highlighted that the furosine level was greater than 300 mg/100 g protein for almost all pasta produced at industrial scale [71]. These values have been found, surprisingly, even in some “artisan pasta”. Moreover, sensory analyses showed that low heat damage (furosine <250 mg/100 g protein) is not a guarantee of good cooking quality. Besides protein content, particle size distribution and, consequently, damaged starch content also greatly affect the furosine levels of pasta samples, even if the same drying cycle is applied [71].

Using wholegrain semolina instead of refined semolina led to increased furosine content, affecting sensory traits. Indeed, pasta with high furosine content (i.e., dried using high-temperature drying cycles) is perceived to be more bitter than pasta with low furosine content (i.e., dried using low-temperature drying cycles) [24]. On the contrary, in pasta made from whole common wheat, drying conditions did not have a significant impact on either taste or flavor (as assessed by descriptive analysis) [68,69].

### 3.9. New Trends in Drying Systems

Most innovations related to the drying stage have aimed at reducing drying times, without affecting pasta quality. In this context, recent work has been carried out on the use of microwaves (either alone or in combination with air drying). The process of drying pasta by microwaves has proven to be very efficient, not only as regards shortening the drying time but also because it is possible to have a final product without fissures, with higher firmness and a lower degree of gelatinization than pasta dried by hot air [72,73,74]. It increased the cooking resistance of pasta as well as its cooking time. Moreover, similar total organic matter values suggest that the cooking quality of samples dried differently was comparable [72,73,74].

More recently, the effect of vacuum drying (where moisture removal from food products occurs under low pressure) on pasta quality has been assessed in semolina pasta at lab scale [75,76]. Compared with conventional drying, vacuum drying is characterized by a lower drying temperature and a higher drying rate (i.e., water evaporation occurs more readily). The enhanced moisture transfer may lead to the prevention of surface barrier formation that causes internal stress within the product. Therefore, the use of vacuum-drying may reduce internal stress and prevent structural deterioration, resulting in better cooking quality (i.e., high water absorption and hardness, low cooking loss and adhesiveness) [75,76]. Moreover, since moisture is removed in the absence of oxygen, oxidative degradations, e.g., browning or fat oxidation, are minimized, resulting in a pasta with a bright yellow color [75,76].

At the industrial level, new drying lines capable of reducing the drying time to about 3 h for long pasta and less than 2 h for short pasta are available, with a significant reduction in the size of the plant. Although the superiority in quality of the product obtained from these systems is claimed by the company that manufactures the drying equipment, no data has been shared with the scientific community. Indeed, most of the studies on processing are mainly conducted by the manufacturers of pasta and/or pasta plants, and thus are subjected to company regulations related to privacy.

## 4. Knowledge Gaps and Perspectives

In this section, the main knowledge gaps related to pasta-making process are summarized.

Since each step in the pasta-making process impacts on the quality of the final product, it is extremely important to know how process variables and pasta properties relate in order to better predict and control product quality. The first steps of the pasta-making process—hydration of semolina and shaping of the dough by extrusion under pressure or roll-sheeting—have so far received less attention than the drying phase. The greater interest in the latter is justified by the modifications (which are well known and quantified) induced by temperatures above 60 °C on both proteins and starch properties and their great impact on pasta quality at both sensory (e.g., texture) and nutritional (e.g., heat damage) levels. A second reason for the apparent minor interest in the hydration and shaping phases is linked to the difficulties that their monitoring entails. In fact, the low humidity (between 30 and 32%) of the mixing system and, consequently, its low degree of smoothness inside the press, makes it difficult to study dough behavior during extrusion. Moreover, the mixture is uneven in temperature and viscosity [77]; these differences can be found not only at the entry of the cylinder towards the die but also at the cross section of the cylinder (in fact, the mass near the walls of the cylinder is colder and with higher consistency than the mass closer to the core of the screw).

Finally, to further complicate observations, process variables (first of all the extrusion pressure) are affected by dough properties (i.e., moisture, temperature, viscosity) and any change in one of the processing variables influences all the others in an interdependent way. In other words, when a parameter changes, the system responds in a very complex way. A further aspect regards the high degree of heterogeneity of extrusion systems due to their different specifications (geometry and pitch of the screw, single- or twin-screw extruder, etc.) that could have different repercussions on the workability of the mixture and on the characteristics of the finished product. Some studies applied prediction models of dough behavior by modifying extrusion variables [77]. However, these works are limited to the study of the process without relating it to the characteristics of the finished product. Finally, there are no studies evaluating the effect of mechanical and structural changes (for example, screw geometry, single- or twin-screw extruder, etc.) on pasta quality.

Moreover, among the studies focusing on the extrusion step, none associates process conditions with the nutritional quality of the finished product, in terms of digestibility and/or the formation of resistant starch. This aspect is left to the reformulation of the product using modified starches or raw materials rich in amylose. In this context, Camelo-Méndez et al. [78] summarizes the effect of different ingredients on the starch digestibility of pasta.

Further gaps come from the pasta quality evaluation side. Most of the studies aimed at understanding the relationship between processing conditions and pasta quality assessed the quality of the final products by evaluating changes in color, cooking loss and texture evaluated by instrumental analysis rather than sensory analysis. Besides requiring less time for the analysis, other factors account for the preference of instrumental tests: (1) a sensory evaluation testing facility should be set up to minimize the interactions occurring between participants; (2) consumer-based sensory evaluation measures liking of foods and requires large numbers of individuals; and (3) descriptive analysis requires trained tasters to evaluate the intensities of attributes found in foods [79].

Finally, most of the studies devoted to understanding the relationships between process conditions and pasta quality considered only semolina as the raw material to be used. Although it is easy to understand the reasons for this choice, worldwide (with the exception of Italy, France and Greece) hard wheat flour is the main raw material used for dry pasta. Indeed, it is widely available and less expensive than durum wheat. However, despite the great interest in describing the bread-making performance of common wheat, it is still unknown what features common wheat should have and what processing parameters should be adopted to obtain dried pasta of desirable quality.

## 5. Conclusions

Dry pasta can be considered an iconic Italian food and is nowadays appreciated around the world for its nutritional and sensory features, as well as for its versatility. Although an established technology, the pasta-making process needs to be optimized, taking into consideration changes in lifestyle and consumer awareness. A healthy diet, resilience and sustainability are the keywords of the era we are living in. Thus, recent interest in fiber-enriched formulations, as well as in underexploited grains necessitates the re-examination, re-thinking and re-adjustment of the conditions currently used for preparing pasta. Attention should be paid to extrusion to ensure the formation of a protein structure that can withstand cooking.

In this context, because the extrusion process is of great interest from an industrial point of view due to its high productivity and versatility, more resources must be allocated to the study and optimization of this phase of the process. More than this would be an opportunity to further the growing interest in alternative raw materials to satisfy increasing nutritional demands and foster environmental, social and economic sustainability, especially in light of future climatic changes that may limit wheat availability and/or deteriorate its quality.

## Figures and Tables

**Table 1:** Parameters affecting dough/pasta quality.

Operation	Aim	Intrinsic Parameters Affecting the Dough/Pasta	Extrinsic Parameters Affecting the Dough/Pasta
Dosing, mixing and kneading	To dose in the right proportions both semolina and water (25–27 parts of water/100 parts of semolina) To hydrate starch and proteins	Semolina particle size Semolina protein, ash, fiber, damaged starch content Enzyme activities Water temperature and residue	Presence of a pre-mixer Vacuum degree
Kneading and shaping by extrusion	To (partially) form gluten networks To knead and give a shape to the dough	Gluten tenacity Dough humidity Dough temperature Dough viscosity	Mixture feeding into the extruder Geometrical characteristics of the screw (length, design, etc.) Extrusion conditions (specific mechanical energy, screw speed, heat regulation system, etc.) Shape of the extruded product Die material Open surface of the die (number and position of the inserts)
Drying	To remove water To assure shape integrity To maintain nutritional quality	Gluten tenacity Starch pasting properties	Air temperature Air relative humidity Drying time
